# Advancing Thermal Energy Storage: Synthesis and Thermal Performance of Silica-Encapsulated Paraffin PCMs

**DOI:** 10.3390/molecules30081698

**Published:** 2025-04-10

**Authors:** Raihana Jannat Adnin, Han-Seung Lee

**Affiliations:** 1 Department of Smart City Engineering, Hanyang University, 1271 Sa 3-dong, Sangnok-gu, Ansan-si 15588, Republic of Korea; jannat12@hanyang.ac.kr; 2 Department of Architectural Engineering, Hanyang University, 1271 Sa 3-dong, Sangnok-gu, Ansan-si 15588, Republic of Korea

**Keywords:** sol–gel synthesis, paraffin, phase change materials, latent heat, thermal energy storage

## Abstract

This study successfully synthesizes SiO_2_-encapsulated nano-phase change materials (NPCMs) via a sol–gel method, using paraffin as the thermal storage medium. The encapsulation process is validated through FTIR, XRD, and XPS analyses, confirming the formation of an amorphous SiO_2_ shell without any chemical interaction between the core and shell. SEM imaging reveals a well-defined core–shell structure with uniform spherical geometry, with the smallest particle size (190 nm) observed in the sample with a 4:1 paraffin/SiO_2_ ratio (PARSI-4). TGA results demonstrate enhanced thermal stability, with thicker SiO_2_ shells effectively protecting against thermal degradation. The DSC analysis indicates that an increased core–shell ratio improves thermal performance, with PARSI-4 exhibiting the highest melting (160.86 J/g) and solidifying (153.93 J/g) enthalpies. The encapsulation ratio (ER) and encapsulation efficiency (EE) have been accomplished at 87.83% and 87.04%, respectively, in the PARSI-4 sample. Thermal cycling tests confirm the material’s long-term stability, with 98.16% enthalpy retention even after 100 cycles. Additionally, leakage resistance tests validate the structural integrity of the encapsulated paraffin, preventing spillage at elevated temperatures. These findings demonstrate the potential of SiO_2_-encapsulated NPCMs for efficient thermal energy storage (TES), making them promising candidates for sustainable and energy-efficient applications.

## 1. Introduction

The rapid expansion of urbanization and the growing global population have led to a significant increase in energy consumption, raising concerns among researchers [1]. According to United Nations projections, the global population is expected to surpass 8.2 billion in 2024 and reach approximately 10.3 billion by the mid-2080s [2,3,4]. This continuous population growth intensifies the demand for energy, resulting in the extensive consumption of non-renewable resources. Consequently, the depletion of these resources has had severe consequences in both urban and rural areas. Despite the limited availability of non-renewable resources, fossil fuels including petroleum, coal, and natural gas contributed 82% of the global energy supply in 2023. Moreover, by 2035, the worldwide demand for fossil fuels is projected to increase by 33% [5,6]. The relentless use of fossil fuels significantly contributes to greenhouse gas emissions, leading to environmental challenges such as pollution, acid rain, global warming, climate change, and ozone layer depletion, all of which pose serious risks to human health and infrastructure [7,8,9]. Studies show that the building, industrial, and transportation sectors are among the largest energy consumers, while buildings alone account for more than 40% of total energy consumption, 35% of greenhouse gas emissions, and two-thirds of the growing electricity demand [10]. A significant portion of this energy is utilized in heating, ventilation, and air conditioning (HVAC) systems to maintain indoor comfort levels. Consequently, rising energy costs have underscored the need for a transition to cleaner, renewable, and more sustainable energy sources [11,12]. Implementing renewable energy solutions is crucial for addressing energy supply and demand challenges while mitigating environmental impacts and reducing reliance on non-renewable resources. In this regard, thermal energy storage (TES) systems have gained significant attention due to their high capacity and flexibility, emerging as an effective strategy for balancing energy supply and demand [13,14]. TES systems can be broadly classified into three major types: sensible heat storage [15], latent heat storage [16], and thermochemical storage [17]. Among these, latent heat thermal energy storage (LHTES) has gained significant attention due to its high energy efficiency, large storage capacity, stable operating temperatures, cost-effectiveness, and compatibility with renewable energy sources [13,18,19].

LHTES systems frequently incorporate phase change materials (PCMs), which efficiently store and release energy through phase transitions. PCMs absorb and discharge thermal energy during melting and solidification, making them highly effective for thermal management applications [20,21,22]. Their superior heat storage and release capabilities, affordability, ease of production, and seamless integration into energy systems have positioned PCMs as a promising solution for energy conservation [1]. Owing to their exceptional thermoregulation properties, PCMs have been widely explored for various applications, including photovoltaic heat management [1,23,24,25], electronic component cooling [26,27,28,29], agricultural greenhouse [30], building energy conservation [31,32,33,34], solar water heating [35,36], and battery technology [37,38].

PCMs are categorized based on their phase transition mechanisms into solid–solid, solid–liquid, solid–gas, and liquid–gas types [39,40]. Among these, solid–liquid PCMs are the most commonly used and can be further classified into organic, inorganic, and eutectic PCMs. Paraffin, a widely utilized organic PCM, offers advantages such as high latent heat capacity, thermal stability, and non-toxicity. However, it also presents challenges, including low thermal conductivity, volume expansion during phase transition, and leakage issues [41,42,43,44]. Encapsulation techniques have been developed to mitigate these challenges by enclosing PCMs within a protective shell. Encapsulation enhances thermal conductivity, prevents leakage, improves structural stability, and optimizes overall PCM performance [45,46]. Based on particle size, encapsulation methods are classified into macro, micro, and nanoencapsulation. Nanoencapsulation has garnered particular interest due to its high surface-area-to-volume ratio, which facilitates improved heat transfer and mechanical stability [47,48]. Additionally, among various shell materials, amorphous SiO_2_ is widely preferred due to its non-toxicity, cost-effectiveness, chemical stability, and superior thermal conductivity [49,50].

However, before integrating encapsulated PCMs into building materials, extensive research and optimization are required to ensure their long-term performance and sustainability. Considering this, this study focuses on encapsulating an alkane with SiO_2_ and assessing its suitability for thermal energy storage systems. This research primarily focuses on solid–liquid organic PCMs, utilizing the sol–gel technique to synthesize nanoencapsulated phase change materials (NPCMs). Paraffin, a commonly used PCM, serves as the core material, while amorphous SiO_2_ forms a protective outer shell, enhancing structural stability and thermal performance. Various characterization techniques have been employed to analyze the synthesized NPCMs, assessing their thermal behavior, stability, and efficiency. This study presents a novel, single-pot sol–gel synthesis approach for the nanoencapsulation of paraffin-based PCMs using amorphous silica as the shell material. The adopted method enables the economic and scalable production of nanostructured silica-encapsulated PCMs with a high surface area and uniform particle distribution. This encapsulation significantly enhances the heat transfer, structural integrity, and leakage resistance of the PCM. The formation of nano-sized silica shells ensures better mechanical stability, overcoming key limitations of conventional PCMs. This innovative methodology offers an advanced, cost-effective solution for efficient thermal energy storage, promoting sustainability across diverse energy applications.

## 2. Results and Discussion

### 2.1. Spectroscopy by FTIR

**Figure 1:**
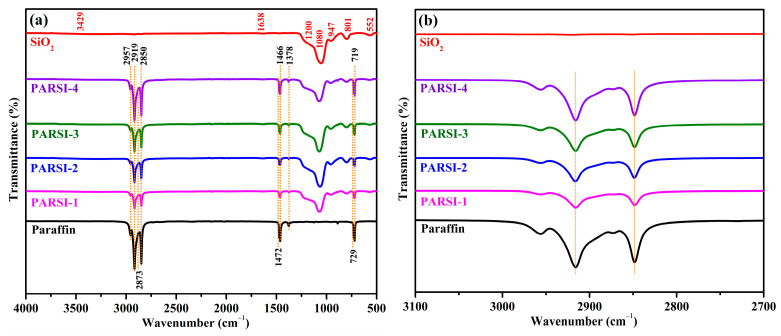
(**a**) FT-IR spectra of paraffin, SiO_2_, PARSI-1, PARSI-2, PARSI-3, and PARSI-4. (**b**) Magnified FT-IR scans at the range of 2700–3100 cm^−1^.

Figure 1a illustrates the FTIR spectra of SiO_2_, bulk paraffin, and SiO_2_-encapsulated NPCMs. The FTIR spectrum of bulk paraffin shows prominent peaks below 3000 cm^−1^ (Figure 1b). The asymmetric stretching of –CH_3_ and –CH_2_ groups appears at absorption peaks of 2957 and 2919 cm^−1^, respectively [51,52]. The symmetric stretching of –CH_3_ and –CH_2_ groups is observed at 2873 and 2850 cm^−1^, respectively [53,54,55]. The peaks at 1472 and 1466 cm^−1^ correspond to the –CH_2_ in-plane deformation and –CH_3_ asymmetric in-plane deformation [49,56,57,58]. The peak at 1378 cm^−1^ is associated with the symmetric bending of the –CH_3_ group [59]. The saturated aliphatic structure of bulk paraffin is further confirmed by the –CH_2_ rocking absorption band and the methyl group of long-chain alkanes, represented by doublet features at 729 cm^−1^ [41,60,61].

In the SiO_2_ spectrum, absorption bands at 552 and 801 cm^−1^ are attributed to the longitudinal optical vibration of Si–O–Si and symmetric stretching vibration of Si–O–Si groups, respectively. The peak at 947 cm^−1^ corresponds to Si–OH bond stretching, and the intensity of this peak reflects the presence of –OH groups on the SiO_2_ surface. The absorption peaks at 1080 and 1200 cm^−1^ are assigned to the out-of-plane and in-plane asymmetric stretching of Si–O–Si, respectively. Additionally, the bending and stretching of water bonds on the SiO_2_ surface are observed near 1638 and 3429 cm^−1^, respectively [62,63,64,65].

For the SiO_2_-encapsulated samples, the observed FTIR peaks align with those of both paraffin and SiO_2_, suggesting the successful formation of the SiO_2_ shell using the sol–gel method. No new peaks are detected, indicating that the interaction between paraffin and SiO_2_ is physical rather than chemical. The FTIR spectra confirms that the encapsulation process preserves the characteristic features of both components without any significant chemical modification. Table 1 summarizes the FTIR wavenumbers and corresponding vibrational modes.

### 2.2. XRD Analysis of the NPCMs

**Figure 2:**
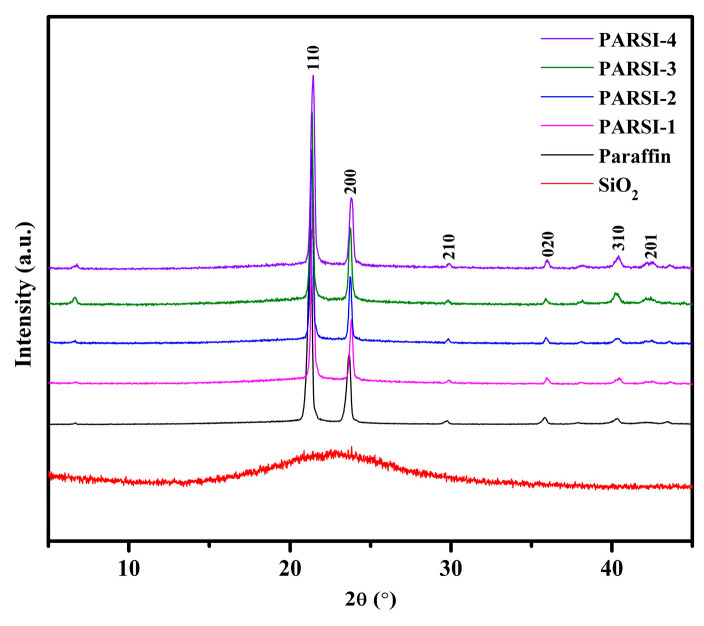
XRD patterns of paraffin, SiO_2,_ and SiO_2_-encapsulated PARSI PCMs.

The X-ray diffraction (XRD) spectra of the synthesized SiO_2_, pure paraffin, and paraffin/SiO_2_ (PARSI) samples are shown in Figure 2. The synthesized SiO_2_ does not exhibit distinct diffraction peaks but instead presents a broad maximum between 2θ values of 15° and 30°, which is characteristic of amorphous SiO_2_ [66,67,68]. In contrast, the SiO_2_-encapsulated PARSI samples display distinct crystalline diffraction peaks superimposed on the broad amorphous SiO_2_ hump. These peaks, located at 2θ angles of 21.38°, 23.84°, 29.80°, 35.92°, 38.12°, 40.36°, and 42.17°, correspond to the crystalline phases of paraffin, as identified in the JCPDS database (No. 0401995). These results are in agreement with previous studies, such as those by Huo et al. [69], Mandal et al. [9], and Zhang et al. [70], which also report the crystalline behavior of paraffin in composite materials. Importantly, no additional diffraction peaks appear in the PARSI samples, confirming that the structure remains a combination of amorphous SiO_2_ and crystalline paraffin, with no chemical interaction occurring between the two components. These findings are further supported by the FTIR analysis, which confirms that the paraffin is encapsulated within the SiO_2_ shell without enduring chemical modification.

### 2.3. XPS Analysis of the NPCMs

**Figure 3:**
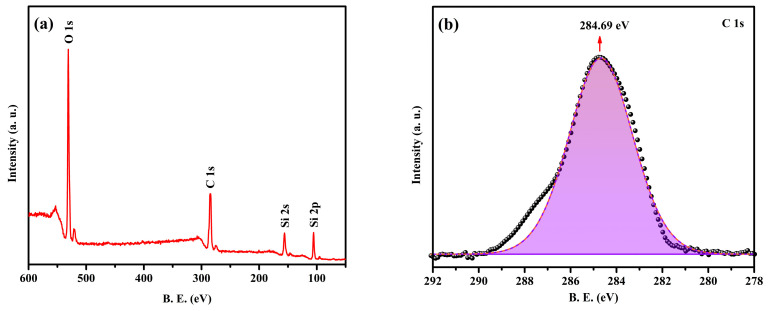

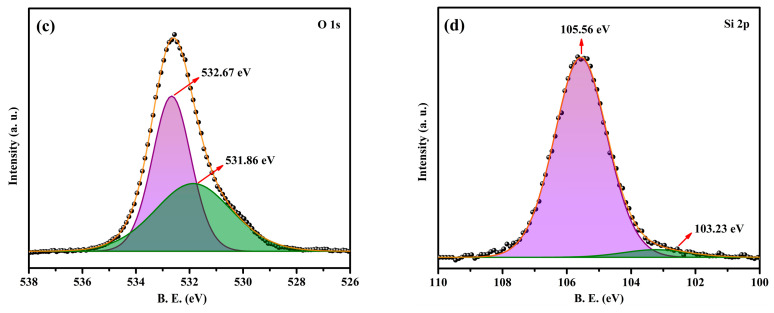
XPS spectra of PARSI-4, (**a**) survey scan, (**b**) C 1s, (**c**) O 1s, and (**d**) Si 2p.

The chemical composition and oxidation states of the elements present in the core (paraffin) and shell (SiO_2_) materials are investigated using XPS, with the analysis focused on the PARSI-4 sample. Figure 3 presents both the survey and high-resolution spectra for the PARSI-4 sample. The deconvoluted high-resolution spectra of the C 1s (Figure 3b), O 1s (Figure 3c), and Si 2p (Figure 3d) regions provide a detailed picture of the interactions between the core and shell materials. In particular, the C 1s peak at 284.69 eV is a clear indicator of the aliphatic nature of paraffin, with the binding energies corresponding to C–C and C–H bonds, which are typical of hydrocarbon structures present in paraffin. This result reaffirms the presence of paraffin in the core material, and its characteristic bonding is consistent with the expected structure of long-chain alkanes. 

The O 1s spectrum provides additional insight into the SiO_2_ shell. The peak at 531.86 eV corresponds to Si–OH bonds, which suggests the presence of hydroxyl groups on the surface of the SiO_2_ particles. These hydroxyl groups are often formed during the sol–gel process used to synthesize SiO_2_, and they play a key role in facilitating the interaction between the SiO_2_ and the paraffin core. The second peak at 532.67 eV, attributed to Si–O–Si bonds, indicates the SiO_2_ network’s typical bonding structure, which is characteristic of amorphous SiO_2_. Additionally, this peak also reflects the presence of chemisorbed water molecules, which are commonly found on the surface of SiO_2_ particles, further emphasizing the material’s hydrophilic nature [71].

The Si 2p spectrum provides two distinct peak fittings for the Si–OH bond at 103.23 eV and the Si–O–Si bond at 105.56 eV. These peaks align well with the literature values for SiO_2_ [72,73], confirming the integrity of the SiO_2_ shell and suggesting no significant chemical modification or transformation of the SiO_2_ during the encapsulation process. The slight shifts in binding energy observed in the XPS spectra are typical for SiO_2_ materials and reflect minor variations in surface chemical environments, which are often due to the presence of hydroxyl groups and adsorbed water molecules on the SiO_2_ surface [72,73]. The XPS result supports the conclusion that the paraffin is physically encapsulated in the SiO_2_ shell, without any chemical alteration or reaction between the core and shell materials, further corroborating the findings in FTIR and XRD.

### 2.4. Morphological Features of the NPCMs

**Figure 4:**
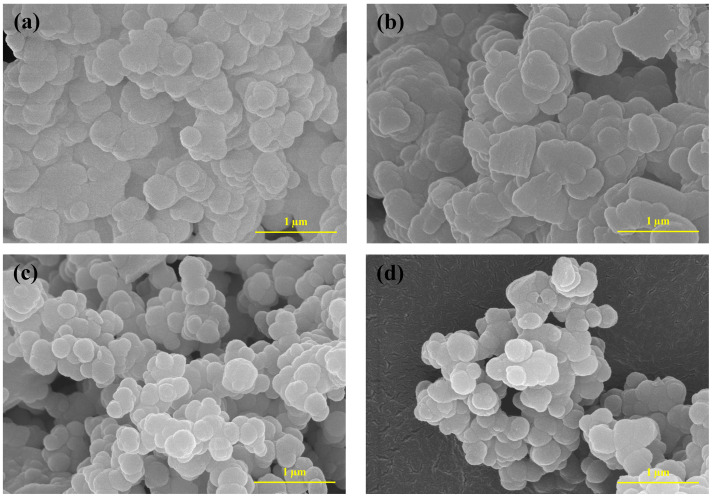
SEM micrographs of (**a**) PARSI-1, (**b**) PARSI-2, (**c**) PARSI-3, and (**d**) PARSI-4.

SEM has been employed to investigate the morphological characteristics of NPCMs, providing crucial insights into their structural integrity, stability, and encapsulation efficiency. The morphology of NPCMs plays a significant role in determining their stability, chemical reactivity, and interfacial compatibility between the core (paraffin) and shell (SiO_2_) materials. The SEM micrographs of the synthesized NPCMs, as illustrated in Figure 4a–d, reveal distinct variations in particle morphology based on composition. Among all the synthesized samples, PARSI-1 exhibits the highest degree of agglomeration, suggesting stronger intermolecular interactions and lower dispersibility. Notably, with an increasing amount of paraffin in the core, the particle size and agglomeration tendency progressively decrease, which can be associated with a more uniform dispersion of paraffin within the SiO_2_ matrix. Furthermore, a greater proportion of paraffin in the core material results in NPCMs with more regular and smoother surface morphologies. This phenomenon can be attributed to the presence of an adequate paraffin volume fraction, which allows for a more uniform distribution of the core material within the SiO_2_ shell, reducing structural irregularities and surface roughness. Despite compositional variations, all NPCMs exhibit a predominantly spherical morphology with relatively homogeneous particle size distributions, further confirming the successful encapsulation process.

The particle size distribution analysis using Image J software (Version 1.54p) reveals that the average particle sizes of the synthesized samples PARSI-1, PARSI-2, PARSI-3, and PARSI-4 are 230 nm, 200 nm, 193 nm, and 190 nm, respectively. The findings also align with previous studies that reported varying particle sizes for similar encapsulation systems. For instance, SiO_2_-encapsulated myristic acid has been reported to exhibit an average particle size of 597 nm [74]. Similarly, SiO_2_-encapsulated paraffin synthesized via the sol–gel method has been reported to have a mean size of 265 nm [75]. In another study, Zhang et al. [76] reported an average particle size of approximately 500 nm for SiO_2_-encapsulated paraffin phase change materials (PCMs), while Fang et al. [77] documented a significantly larger average particle size of 2 µm for SiO_2_-encapsulated n-tetradecane.

The observed discrepancies in particle size across different studies can be attributed to variations in synthesis parameters, including the nature of the precursor materials, reaction conditions, surfactant use, and emulsification techniques. The present study’s results, which demonstrate an average particle size of ~200 nm, suggest a well-controlled encapsulation process with a high degree of uniformity, making the synthesized NPCMs suitable for applications requiring nano-sized thermal energy storage materials. The SEM results, in conjunction with complementary characterization techniques such as FTIR, XRD, and XPS, conclusively validate the successful encapsulation of paraffin within the SiO_2_ shell, thereby confirming the structural and morphological integrity of the synthesized NPCMs.

To understand the elements present in the PARSI samples, EDS analyses are carried out. The obtained mass percentages of C, O, and Si from the EDS analyses are presented in Table 2. It can be seen from the EDS data that with increasing core materials, the C percentage increases, and the content of Si and O decreases.

### 2.5. Thermal Stability Evaluation of the NPCMs

**Figure 5:**
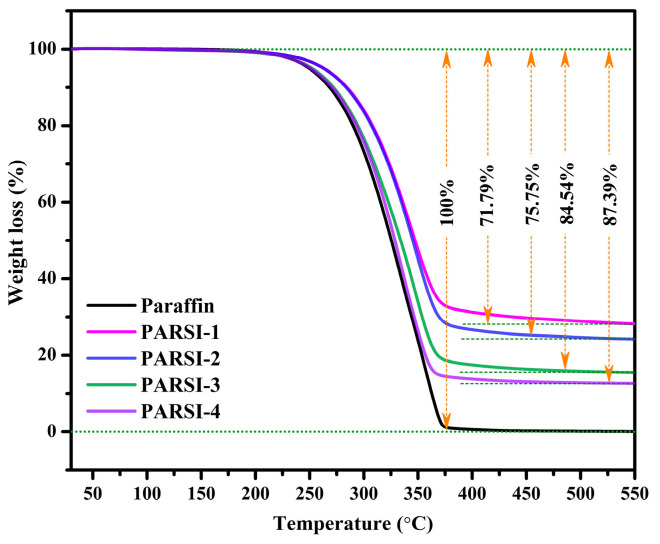
TGA thermogram of pure paraffin and PARSI NPCMs.

The ability of the synthesized NPCMs to maintain structural and functional integrity at elevated temperatures is essential for their practical applications in TES systems. To assess the thermal degradation behavior of pure paraffin and synthesized NPCMs, TGA is conducted within a temperature range of 30–550 °C, and the thermograms are presented in Figure 5. The TGA results indicate that all the synthesized NPCM samples remain thermally stable up to 185 °C without any significant mass loss, suggesting the absence of premature degradation or volatilization at lower temperatures. However, beyond this threshold, a gradual thermal decomposition process begins. The onset degradation temperature of pure paraffin is observed at approximately 186.5 °C, followed by complete degradation near 379 °C. In contrast, all the synthesized NPCMs exhibit higher onset degradation temperatures compared to pure paraffin, confirming the protective effect of the SiO_2_ shell. Specifically, the onset degradation temperatures for the encapsulated samples are determined to be 200.59 °C, 197.68 °C, 192.71 °C, and 190.77 °C for PARSI-1, PARSI-2, PARSI-3, and PARSI-4, respectively. The observed enhancement in thermal stability is due to the formation of a stable SiO_2_ shell around the paraffin core, which serves as a structural barrier, mitigating the direct exposure of the core material to external heat sources and reducing the rate of evaporation [76,78].

The extent of thermal degradation is calculated by the weight loss percentages of the NPCM samples. The weight loss values are 71.79%, 75.75%, 84.54%, and 87.39% for PARSI-1, PARSI-2, PARSI-3, and PARSI-4, respectively. This trend suggests that NPCMs with a higher SiO_2_ content exhibit lower weight loss percentage, reinforcing the role of the shell material in enhancing thermal stability. The presence of a thicker SiO_2_ shell provides superior encapsulation efficiency, minimizing paraffin leakage and evaporation at elevated temperatures. The encapsulation effect is further corroborated by previous studies, where a similar enhancement in thermal stability is observed for phase change materials (PCMs) encapsulated with calcium carbonate and other inorganic shells [10,79,80].

Additionally, a comparative analysis of degradation delays among the encapsulated samples reveals that the thermal decomposition of NPCMs is delayed by approximately 4.27 °C, 6.21 °C, 11.18 °C, and 14.09 °C for PARSI-4, PARSI-3, PARSI-2, and PARSI-1, respectively. Therefore, the TGA results conclusively demonstrate that the encapsulation of paraffin with SiO_2_ not only enhances heat transfer but also delays the onset of degradation. The increasing core–shell ratio contributes to the formation of a thicker shell, which acts as a protective layer against thermal decomposition. This enhanced thermal performance makes SiO_2_-encapsulated NPCMs promising candidates for high-temperature thermal energy storage applications.

### 2.6. Heat Storage Capability of the NPCMs

**Figure 6:**
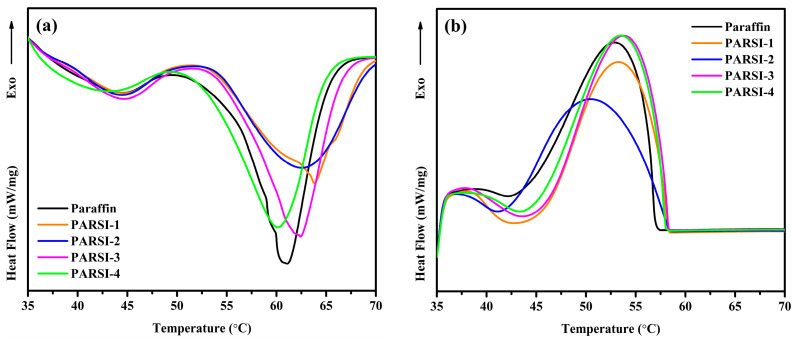
DSC curves of (**a**) melting, and (**b**) solidification of pure paraffin, PARSI samples.

DSC is employed to investigate the physicochemical properties of the synthesized nanoparticle phase change materials (NPCMs), including phase transition temperatures and phase change enthalpies. The DSC curves of pure paraffin and its encapsulated derivatives (NPCMs) are presented in Figure 6, while the corresponding thermal parameters are summarized in Table 3. The DSC results indicate that the melting temperature (T_m_) and solidifying temperature (T_s_) of pure paraffin are 61.08 °C and 53.09 °C, respectively. The corresponding melting (ΔH_m_) and solidifying (ΔH_s_) enthalpies are 183.14 J/g and 178.51 J/g, respectively. In contrast, among the synthesized NPCMs, the lowest melting and solidifying enthalpies are observed in PARSI-1, with values of 130.13 J/g and 126.71 J/g, respectively. However, the enthalpies (ΔH_m_ and ΔH_s_) increase with a higher core–shell ratio. The melting enthalpy (ΔH_m_) values for PARSI-2, PARSI-3, and PARSI-4 are recorded as 137.63 J/g, 152.98 J/g, and 160.86 J/g, respectively, whereas their respective solidifying enthalpy (ΔH_s_) values are 131.57 J/g, 147.44 J/g, and 153.93 J/g. At a higher core–shell ratio, particularly in PARSI-4, the increased paraffin content enhances heat storage in the core, leading to an improved thermal storage capacity. The results suggest that the latent heat storage capability of NPCMs depends on the proportion of paraffin encapsulated within the SiO_2_ shell [10].

An interesting observation is found in the decreasing trend of T_m_. Initially, the T_m_ found for paraffin is at 61.08 °C. The same has been increased in PARSI-1 to 63.83 °C and consistently reduced with increasing content of core materials. Finally, for PARSI-4 NPCM, the T_m_ is found at 60.05 °C. This can be ascribed as a thinner SiO_2_ shell, associated with a higher paraffin content, reduces the confinement effect of the paraffin molecules inside the core, thereby facilitating better phase change properties. Conversely, a lower core–shell ratio results in a thicker SiO_2_ shell, which restricts the movement of the core material and affects heat absorption and release [72,81,82]. During the melting process of both pure paraffin and the NPCMs, structural relaxation in the long-chain paraffin molecules leads to the appearance of a small endothermic peak indicative of solid–solid phase transitions. Similar observations are also observed in the cooling curves of all the samples. Additionally, weak interactions between the paraffin core and the SiO_2_ shell such as capillary forces, hydrogen bonding, and surface tension, further contribute to the decrease in T_m_ of NPCMs [83].

To comprehensively evaluate the thermal energy storage performance of the synthesized NPCMs, the encapsulation ratio (*ER%*), encapsulation efficiency (*EE%*), and thermal storage capability (*η%*) are calculated using the following equations [10,72,80,84,85]:

(1) $$ER\%=\frac{∆H_{m,\;NPCM}}{∆H_{m,\;PCM}}\times 100$$

(2) $$EE\%=\frac{∆H_{m,NPCM}+∆H_{s,NPCM}}{∆H_{m,PCM}+∆H_{s,PCM}}\times 100$$

(3) $$\eta \%=\frac{\frac{∆H_{m,NPCM}+∆H_{s,NPCM}}{ER}}{∆H_{m,PCM}+∆H_{s,PCM}}\times 100=\frac{EE}{ER}\times 100$$

where Δ*H_m_,_PCM_* and Δ*H_s_,_PCM_* represent the melting and solidification enthalpies of pure paraffin, respectively, while Δ*H_m_,_NPCM_* and Δ*H_s_,_NPCM_* denote the melting and solidification enthalpies of the synthesized NPCMs, respectively. The calculated values of *ER%*, *EE%*, and *η%* are summarized in Table 3. The encapsulation ratio (*ER*) reflects the effective confinement of paraffin within the SiO_2_ shell and represents the ratio of the PCM core to the total mass of the NPCMs. The *ER* values for PARSI-1, PARSI-2, PARSI-3, and PARSI-4 are determined to be 71.05%, 75.15%, 83.53%, and 87.83%, respectively. Furthermore, the encapsulation efficiency (*EE*) is found to be 71.02%, 74.44%, 83.08%, and 87.04% for PARSI-1, PARSI-2, PARSI-3, and PARSI-4, respectively. Notably, both the encapsulation ratio and encapsulation efficiency increase as the core–shell ratio rises, with PARSI-4 exhibiting the highest *ER%* and *EE%* values, reaching 87.83% and 87.04%, respectively. This finding suggests that enhancing the core–shell ratio significantly improves encapsulation performance. The thermal storage and release efficiency of the NPCMs is further evaluated using Equation (3). The thermal storage capability (*η*) is calculated as 99.95%, 99.05%, 99.46%, and 99.10% for PARSI-1, PARSI-2, PARSI-3, and PARSI-4, respectively. These results indicate that all encapsulated PCMs exhibit excellent energy storage and release capabilities during phase transitions, demonstrating their potential for thermal energy storage applications.

Given the scarcity of research focused on encapsulation techniques for PCMs, efforts have been made to compile encapsulation ratios (*ER%*) and encapsulation efficiencies (*EE%*) from the available literature, as summarized in Table 4. The higher *ER%* and *EE%* observed for PARSI-4 underscore its suitability for eco-friendly and advanced thermal energy applications, emphasizing its promising role in sustainable energy storage solutions.

### 2.7. Thermal Cycle Endurance of the NPCMs

**Figure 7:**
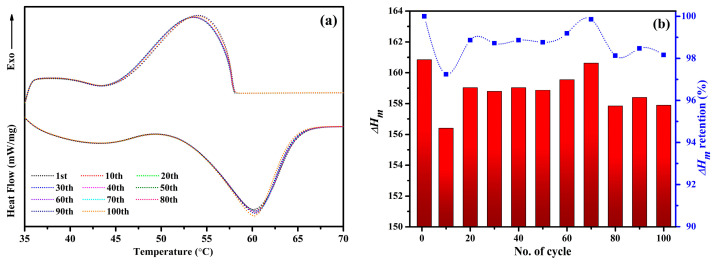
(**a**) DSC thermal cycles (100 cycles) and (**b**) melting enthalpy retention during thermal cycles of PARSI-4.

The evaluation of thermal stability and long-term performance is essential for PCMs to ensure their viability in TES applications. To examine the thermal reliability of the synthesized NPCMs, DSC thermal cycling tests are conducted on the PARSI-4 sample, subjecting it to 100 consecutive heating and cooling cycles. The corresponding thermogram is illustrated in Figure 7a, presenting the DSC curves for the initial cycle, and every tenth cycle. The variations in melting enthalpy with their retention percentages over the thermal cycles are presented together in Figure 7b. The results reveal a consistent trend in thermal behavior across multiple cycles. Initially, the melting enthalpy (∆H_m_) of PARSI-4 is measured at 160.86 J/g. After undergoing 100 thermal cycles, the melting enthalpy slightly decreases to 157.89 J/g, maintaining approximately 98.16% of its initial value. The DSC curves exhibit substantial consistency, suggesting that the encapsulated PCM maintains stable thermal performance over prolonged use.

These findings confirm that the encapsulated paraffin within the SiO_2_ shell retains its heat storage capacity with minimal degradation, reinforcing its mechanical and chemical stability. The negligible variation in enthalpy retention indicates that the encapsulation process effectively enhances the durability of the PCM, making it a promising candidate for long-term thermal energy storage applications.

### 2.8. Leakage Resistance Performance

**Figure 8:**
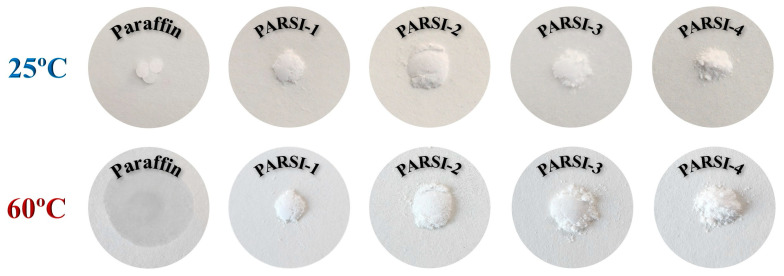
Leakage resistance performance of paraffin and PARSI samples at room temperature (25 °C) and 60 °C.

Leakage resistance is a crucial parameter in determining the structural integrity, durability, and practical applicability of PCMs. It directly impacts the stabilities of the shell material and the overall TES efficiency. To assess the leakage-proof performance, paraffin and all synthesized NPCMs are evaluated at ambient conditions (25 °C) and at an elevated temperature of 60 °C, which exceeds the melting point of pure paraffin. The digital images of the samples, depicted in Figure 8, highlight the distinct differences in leakage behavior between pure paraffin and NPCMs. As expected, when pure paraffin is heated beyond its melting point, it undergoes complete liquefaction, leading to immediate leakage. This uncontrolled phase transition reduces its thermal efficiency and limits its practical applications. In contrast, the NPCM samples exhibit significantly enhanced leakage resistance, maintaining their structural integrity even at 60 °C. The presence of a SiO_2_ shell effectively shields the paraffin core, forming a protective barrier that prevents leakage and ensures stable phase transition behavior.

The superior leakage resistance of SiO_2_-encapsulated paraffin confirms that the SiO_2_ shell plays a pivotal role in improving the shape stability and long-term durability of the material. This enhanced structural integrity is essential for practical applications, particularly in TES systems where material containment and thermal reliability are important. These findings emphasize the effectiveness of SiO_2_ encapsulation in mitigating phase change leakage, ensuring that NPCMs can be utilized efficiently in real-world thermal management applications.

## 3. Materials and Methods

### 3.1. Materials

Paraffin wax (C_n_H_2n+2_) with a melting point of 56–58 °C and a purity of 99 wt.% was selected as the core material for thermal energy storage. Cetyltrimethylammonium bromide (CTAB, 99% purity) was employed as an oil–water emulsifier, while tetraethoxysilane (TEOS) served as the SiO_2_ precursor solution. All three materials were obtained from Daejung Chemical & Metals Co., Ltd., Siheung, South Korea. Absolute ethyl alcohol (EA, 99.5% purity) was used as a solvent, and ammonia water (27 wt.%, NH_4_OH) acted as a catalyst, both of which were procured from Duksan Pure Chemicals, Ansan, South Korea. All chemicals used in the study were of reagent grade and were utilized without any additional purification.

### 3.2. Encapsulation Technique of Paraffin@SiO_2_ NPCMs

Silicon-based nanoencapsulated paraffin was synthesized using a simple one-pot sol–gel method within an oil–water emulsion. In this process, varying amounts of paraffin were placed in a conical flask along with a fixed quantity of the SiO_2_ precursor, TEOS. The encapsulation involved three key steps: emulsion preparation, gel formation, and polycondensation. Initially, the oil–water (O/W) emulsion was created by dissolving different amounts of paraffin in 50 mL of absolute EA while stirring magnetically at 1000 rpm and heating to 60 °C. After 20 min of continuous emulsification, when the solution became transparent, 1 g of CTAB was introduced to facilitate micelle formation. Subsequently, 1 mL of TEOS was added dropwise, and the solution was maintained under constant stirring for gel formation. Then, 6 mL of 27% NH_4_OH was added, allowing interfacial hydrolysis and polycondensation of TEOS to occur over 3 h. The resulting solution was centrifuged at 4500 rpm to isolate the nanoencapsulated PCMs (NPCMs), which were then washed with ethanol and distilled water 5 times to remove impurities. Finally, the purified NPCMs were dried in an oven at 50 °C for 24 h. The precise quantities of chemicals used in the encapsulation process are detailed in Table 5.

### 3.3. Mechanism of Encapsulation

In this simple one-pot encapsulation approach, the SiO_2_ precursor, TEOS, undergoes polycondensation, leading to the formation of long SiO_2_ chains. To stabilize the paraffin/ethanol emulsion, the cationic surfactant CTAB is introduced, ensuring the separation of hydrophobic and hydrophilic regions. At this stage, the hydrophobic segments of CTAB adhere to the paraffin core, preventing its interaction with water, while the hydrophilic segments remain connected to water molecules, creating a stable emulsion.

**Figure 9:**
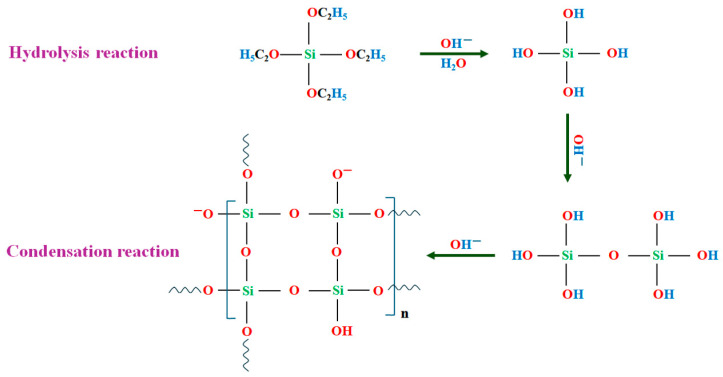
General scheme for the processes of hydrolysis and condensation of TEOS.

Before the condensation reaction begins, TEOS hydrolyzes upon introduction, forming orthosilicic acid [Si(OH)_4_], which then migrates to the outer layer of the paraffin droplets. The polycondensation reaction is initiated by the catalyst, NH_4_OH, leading to the aggregation of hydrolyzed TEOS molecules into SiO_2_ clusters. These clusters form a protective SiO_2_ shell around the paraffin in the presence of CTAB, completing the encapsulation process. A detailed overview of this process is illustrated in Figure 9.

### 3.4. Characterization Techniques

#### 3.4.1. Microscopy

The morphology and elemental distribution of the nanoencapsulated phase change materials (PCMs) were analyzed using a field emission scanning electron microscope (FE-SEM; Hitachi S-4800, Tokyo, Japan) at an accelerating voltage of 15 kV. To prevent dark and blurred images caused by the low conductivity of SiO_2_, all samples were coated with a thin layer of platinum (Pt), effectively minimizing charging effects and enhancing image clarity. The chemical constituents of the PARSI samples are investigated using energy-dispersive X-ray spectroscopy (EDS) attached to the SEM.

#### 3.4.2. X-Ray Diffraction

X-ray diffraction (XRD) analysis was performed using a D/MAX-2500 (Rigaku, Tokyo, Japan) to determine the phases and crystalline structure of both the bulk and core materials of the encapsulated PCMs. The analysis utilized Cu-Kα radiation (λ = 1.541 Å) under operating conditions of 25 kV and 100 mA. XRD patterns were recorded over a 2θ range of 5°–50° with a scanning rate of 4°/min and a step size of 0.02°.

#### 3.4.3. Spectroscopy

Attenuated Total Reflectance Fourier transform infrared spectroscopy (ATR-FTIR) was conducted using a Perkin Elmer UATR Two (Shelton, CT, USA) to analyze the functional groups present in bulk paraffin, SiO_2_, and synthesized NPCMs, as well as to examine their molecular interactions. The spectra were recorded within the wavenumber range of 500 cm^−1^ to 4000 cm^−1^.

To further analyze the elemental composition and oxidation states of the encapsulated PCMs, X-ray photoelectron spectroscopy (XPS) was performed using a Scienta Omicron R300 (Denve, CO, USA). Aluminum-Kα (hv = 1486.6 eV) radiation serves as the energy source for acquiring both full and fine spectra. The obtained XPS spectra were calibrated using the C 1s aliphatic peak of contaminant carbon, which has a binding energy (B.E.) of 284.6 eV. Background subtraction was carried out using the Shirley method, and high-resolution XPS peaks for individual elements were deconvoluted using CASA XPS software (Version 2.3.17). Optimal peak fitting was achieved through a combination of Lorentzian and Gaussian functions.

#### 3.4.4. Thermal Analysis

Thermal stability and decomposition behavior of both pure paraffin and synthesized NPCMs were evaluated using thermogravimetric analysis (TGA) with a PerkinElmer STA 6000 (Waltham, MA, USA) at a constant heating rate of 10 °C/min, covering a temperature range of 30–550 °C. The thermal properties of the paraffin and NPCMs, such as phase change temperatures (crystallization/melting), phase change latent heats, and thermal reliability (thermal cycling), were measured using a differential scanning calorimeter (DSC-Q 20, TA Instruments, New Castle, DE, USA). All measurements were performed under a nitrogen atmosphere at heating or cooling rates of 10 °C/min, with a temperature range from 35 °C to 70 °C.

#### 3.4.5. Leakage Test

The leakage resistance is a crucial performance indicator for PCMs. To evaluate this, pure paraffin and NPCMs were placed on Whatman filter papers and heated on a hot plate to 60 °C for 30 min. The samples were then visually inspected for any potential leakage, indicated by stains on the filter paper.

## 4. Conclusions

In this study, SiO_2_-encapsulated phase change materials (NPCMs) have been successfully synthesized using the sol–gel method for thermal energy storage applications. The key findings of the research include the following:

- FTIR and XRD analyses confirm the presence of Si–O–Si and Si–OH bonds, indicating the successful encapsulation of paraffin within the SiO_2_ shell without any chemical interaction between the core and shell materials. The amorphous nature of the SiO_2_ shell is validated by XRD results.
- The XPS analysis further affirms the presence of O 1s and Si 2p peaks, supporting the successful encapsulation of paraffin within the SiO_2_ shell and corroborating the FTIR and XRD findings.
- The SEM analysis reveals a well-defined core–shell structure with uniform spherical particles, and the particle sizes vary depending on composition. The smallest average particle size of 190 nm is observed for the PARSI-4 sample.
- The TGA results indicate that SiO_2_ encapsulation provides effective protection against thermal degradation, where thicker SiO_2_ shells offer enhanced thermal stability, ensuring better thermal resistance.
- The thermal properties of NPCMs improve with an increasing core–shell ratio, with the PARSI-4 sample exhibiting the highest melting (160.86 J/g) and solidifying (153.93 J/g) enthalpies, demonstrating superior heat storage capacity.
- The encapsulation ratio and encapsulation efficiency increase with the core–shell ratio, where the PARSI-4 sample achieves the highest *ER* (87.83%) and *EE* (87.04%), ensuring efficient paraffin holding within the SiO_2_ shell.
- After 100 DSC thermal cycles, the PARSI-4 sample retains 98.16% of its initial enthalpy, highlighting its excellent thermal reliability and long-term applicability for thermal energy storage systems.
- The SiO_2_ shell effectively prevents paraffin leakage, ensuring shape stability and durability even at elevated temperatures, demonstrating the successful encapsulation and practical feasibility of NPCMs in thermal energy storage applications.
- This novel single-pot sol–gel synthesis enables the economical fabrication of nano-sized silica-encapsulated paraffin with a high surface area, enhancing heat transfer, thermal stability, and storage efficiency. This innovative approach offers a scalable solution for advanced TES applications, supporting sustainable energy goals by improving PCM performance and reducing dependence on fossil fuels.

## Figures and Tables

**Table 1:** FTIR wavenumbers and their corresponding vibrational modes.

Wavenumber (cm^−1^)	Vibration Type	Assignment
2957	asymmetric stretching	–CH_3_
2919	asymmetric stretching	–CH_2_
2873	symmetric stretching	–CH_3_
2850	symmetric stretching	–CH_2_
1472	in plane deformation	–CH_2_
	asymmetric in-plane deformation	–CH_3_
1466	in plane deformation	–CH_2_
	asymmetric in-plane deformation	–CH_3_
1378	symmetric carbon-hydrogen bending absorption	–CH_3_
719/729	rocking	–CH_2_
3429	stretching	–OH
1638	bending	–OH
1200	in-plane asymmetric stretching	Si–O–Si
1080	Out of plane asymmetric stretching	Si–O–Si
947	stretching	Si–OH
801	symmetric stretching	Si–O–Si
552	longitudinal optical vibration	Si–O–Si

**Table 2:** Presence of elements (%) in SiO_2_-encapsulated PARSI samples.

Sample ID	Elements (Mass Norm.%)		
	C	O	Si
PARSI-1	43.54	39.40	17.06
PARSI-2	48.89	37.17	13.94
PARSI-3	52.34	35.11	12.55
PARSI-4	60.09	30.13	9.78

**Table 3:** Thermal parameters and encapsulation characteristics of pure paraffin and NPCMs derived from DSC analysis.

Sample ID	T_m_ (°C)	∆H_m_ (J/g)	T_s_ (°C)	∆H_s_ (J/g)	*ER%*	*EE%*	*η %*
Paraffin	61.08 ± 0.5	183.14 ± 1.2	53.09 ± 0.7	178.51 ± 1.4	-	-	-
PARSI-1	63.83 ± 0.4	130.13 ± 1.6	53.30 ± 0.3	126.71 ± 1.7	71.05 ± 0.41	71.02 ± 0.40	99.95
PARSI-2	62.68 ± 0.6	137.63 ± 0.9	50.70 ± 0.2	131.57 ± 2.1	75.15 ± 0.02	74.44 ± 0.31	99.05
PARSI-3	62.42 ± 0.1	152.98 ± 1.4	53.87 ± 0.6	147.49 ± 1.8	83.53 ± 0.22	83.08 ± 0.28	99.46
PARSI-4	60.05 ± 0.3	160.86 ± 1.5	53.50 ± 0.3	153.93 ± 1.1	87.83 ± 0.25	87.04 ± 0.09	99.10

**Table 4:** Encapsulation ratio and efficiency values of PARSI-4 compared with the values in the literature.

Serial No.	Core Material	Shell Material	∆H_m_ (J/g)	∆H_s_ (J/g)	*ER%*	*EE%*	Refs.
1.	Paraffin	SiO_2_	94.4	93.2	74.51	75.58	[70]
2.	Paraffin	SiO_2_	161.4	158.1	-	80	[86]
3.	Paraffin	SiO_2_	156.6	158.1	78.2	78.4	[87]
4.	Paraffin	SiO_2_	117.7	110.8	61.88	61.84	[88]
5.	Paraffin	SiO_2_	189.4	-	-	81.3	[89]
6.	Paraffin	SiO_2_	191.67	188.84	75.97	75.82	[76]
7.	Paraffin	SiO_2_	91.9	89.7	-	52.7	[90]
8.	Paraffin	SiO_2_	79.2	83.5	42.33	44.13	[78]
9.	Paraffin	SiO_2_	13	-	11	-	[91]
10.	Paraffin	SiO_2_	45.5	43.8	31.7	31.1	[92]
11.	Paraffin	SiO_2_	94.4	93.2	74.51	75.58	[70]
12.	Paraffin	SiO_2_–TiO_2_	93.70	-	39.80	-	[93]
13.	Heptadecane	SiO_2_	60.25	61.37	30.94	30.48	[94]
14.	Octadecane	SiO_2_	73.52	72.18	35.62	35.18	[94]
15.	Octadecane	SiO_2_	87.46	84.89	41.83	41.45	[95]
16.	Octadecane	SiO_2_	123.00	125.40	57.50	57.70	[96]
17.	Octadecane	SiO_2_	109.9	103.4	53.8	-	[97]
18.	Octadecane	SiO_2_	109.5	98.85	51.5	49.3	[98]
19.	Nonadecane	SiO_2_	74.78	80.79	41.12	40.64	[94]
20.	Eicosane	SiO_2_	81.21	78.63	32.97	32.51	[94]
21.	Eicosane	SiO_2_	170.20	169.60	71.78	71.72	[99]
22.	Docosane	SiO_2_	181.5	-	78.6	80.8	[100]
23.	Paraffin	SiO_2_	160.86	153.93	87.83	87.04	This study

**Table 5:** Names of NPCMs with different core–shell ratios and the amounts of precursors.

Sample	Core/Shell Ratio	Ethanol (mL)	Paraffin (g)	TEOS (mL)	CTAB (g)	Ammonia Water (mL)
PARSI-1	1:1	50	1	1	1	6
PARSI-2	2:1		2			
PARSI-3	3:1		3			
PARSI-4	4:1		4			

## Data Availability

The data presented in this study are available on request from the corresponding author due to privacy.
